# Patterns of Genomic Instability in Interspecific Yeast Hybrids With Diverse Ancestries

**DOI:** 10.3389/ffunb.2021.742894

**Published:** 2021-10-12

**Authors:** Devin P. Bendixsen, David Peris, Rike Stelkens

**Affiliations:** ^1^Population Genetics Division, Department of Zoology, Stockholm University, Stockholm, Sweden; ^2^Section for Genetics and Evolutionary Biology, Department of Biosciences, University of Oslo, Oslo, Norway; ^3^Department of Health, Valencian International University, Valencia, Spain

**Keywords:** yeast, hybridization, aneuploidy, introgression, genome instability, loss of heterozygosity, *Saccharomyces*

## Abstract

The genomes of hybrids often show substantial deviations from the features of the parent genomes, including genomic instabilities characterized by chromosomal rearrangements, gains, and losses. This plastic genomic architecture generates phenotypic diversity, potentially giving hybrids access to new ecological niches. It is however unclear if there are any generalizable patterns and predictability in the type and prevalence of genomic variation and instability across hybrids with different genetic and ecological backgrounds. Here, we analyzed the genomic architecture of 204 interspecific *Saccharomyces* yeast hybrids isolated from natural, industrial fermentation, clinical, and laboratory environments. Synchronous mapping to all eight putative parental species showed significant variation in read depth indicating frequent aneuploidy, affecting 44% of all hybrid genomes and particularly smaller chromosomes. Early generation hybrids with largely equal genomic content from both parent species were more likely to contain aneuploidies than introgressed genomes with an older hybridization history, which presumably stabilized the genome. Shared k-mer analysis showed that the degree of genomic diversity and variability varied among hybrids with different parent species. Interestingly, more genetically distant crosses produced more similar hybrid genomes, which may be a result of stronger negative epistasis at larger genomic divergence, putting constraints on hybridization outcomes. Mitochondrial genomes were typically inherited from the species also contributing the majority nuclear genome, but there were clear exceptions to this rule. Together, we find reliable genomic predictors of instability in hybrids, but also report interesting cross- and environment-specific idiosyncrasies. Our results are an important step in understanding the factors shaping divergent hybrid genomes and their role in adaptive evolution.

## Introduction

Hybridization generates novel genomic combinations by merging divergent genomes, each of which has been uniquely refined by natural selection and stochastic processes through evolutionary time. This rapid blend of divergent DNA results in a myriad of genomic consequences and often leads to genomic instability in *Saccharomyces* yeasts (Dunn et al., 2013; Morard et al., 2020; Steensels et al., 2021) and other organisms (Dion-Côté and Barbash, 2017; Gibeaux et al., 2018). Recent years have seen considerable progress in identifying and describing genomic features of hybrids. For instance, it was recently found that genetic outcomes of hybridization are surprisingly repeatable in crosses between closely related species (Langdon et al., 2021; Moran et al., 2021). However, there are many open questions about the predictability of hybrid genomic architecture, the prevalence of genomic instabilities, and their evolutionary relevance. Can we predict hybrid genomes from parental genomic, demographic, or ecological features at all?

Some outcomes of hybridization are well-known. Hybridization between genetically distant yeast species usually causes significant genomic dysfunction. As a result, only a small fraction (<1%) of the spores of interspecific *Saccharomyces* F1 hybrids are viable and fertile. This lucky subset however can reproduce asexually and/or sexually and may be evolutionarily successful, in some cases even establish new lineages that are reproductively isolated from the parents. Reproductive isolation of intra- and interspecific hybrids can be mediated by ecology, e.g., through adaptation to a new niche (Leducq et al., 2016) or by endogenous factors, e.g., through allopolyploidy or chromosomal rearrangements preventing backcrosses (Charron et al., 2014). Whole genome duplication (WGD) has been shown to help circumvent severe fitness loss in the F2 hybrid generation (Marsit et al., 2021). However the F1 spores of allotetraploid hybrids are not able to mate with each other or a parent because their heterozygosity at the mating type locus renders them sterile (Pfliegler et al., 2012). Recently, two genetic mechanisms have been shown to restore fertility in normally sterile crosses, giving hybrids a route to meiotic recombination and adaptation. This includes return-to-growth (RTG) in intraspecific *Saccharomyces cerevisiae* crosses (Mozzachiodi et al., 2020), and massive loss of heterozygosity (LOH) (D'Angiolo et al., 2020).

But before hybrid genomes are stable and established, they often undergo wholesale, rapid changes (Sipiczki, 2008, 2018; Pfliegler et al., 2012; Lopandic, 2018; Steensels et al., 2021). Due to the failure of homeologeous chromosomes to pair, and antirecombination preventing regular chromosomal segregation in hybrid meiosis (Hunter et al., 1996; Rogers et al., 2018; Bozdag et al., 2021), the gametes (spores) of F1 hybrids are often aneuploid (reviewed in Gilchrist and Stelkens, 2019). Aneuploidy can cause the dysregulation of gene expression, which is often detrimental to the cell (Pavelka et al., 2010a,b; Sheltzer et al., 2012; Dürrbaum and Storchová, 2016). Thus, most extra chromosomes are short-lived and shed in consecutive mitotic divisions. However, some aneuploidies have been shown to be well tolerated in wild strains of *S. cerevisiae* through gene dosage compensation (Gasch et al., 2016; Tsai and Nelliat, 2019; Scopel et al., 2021) and some specific disomies, trisomies or segmental aneuploidies can in fact provide quick adaptive solutions, especially when cells face environmental stress (e.g., heat or antibiotics) (Rancati et al., 2008; Selmecki et al., 2009; Chen et al., 2012; Yona et al., 2012; Morard et al., 2019). The higher the relative fitness effect of a specific aneuploidy, the more likely this aneuploidy will remain a more permanent feature of the hybrid genome, but so far these effects remain unquantified in interspecific yeast hybrids.

Here, for the first time, we test for patterns of chromosomal mis-segregation across a diverse set of hybrid genomes with different species ancestries, evolutionary histories, and ecological niches. We predict aneuploidies and their adaptive relevance to vary with the parental genomic background of the hybrid cross and with environmental conditions. Specifically, we expect more genetically divergent parents to generate more aneuploid offspring. We also predict that the more skewed the parental genomic contributions are in a hybrid genome, the fewer aneuploidies it will carry, because overall sequence similarity increases, mitigating the risk for chromosome missegregation. Consequently, we predict “older” introgressed genomes to be more euploid and stable overall, as they had more time for postzygotic genomic processes after hybridization.

**Figure 1:**
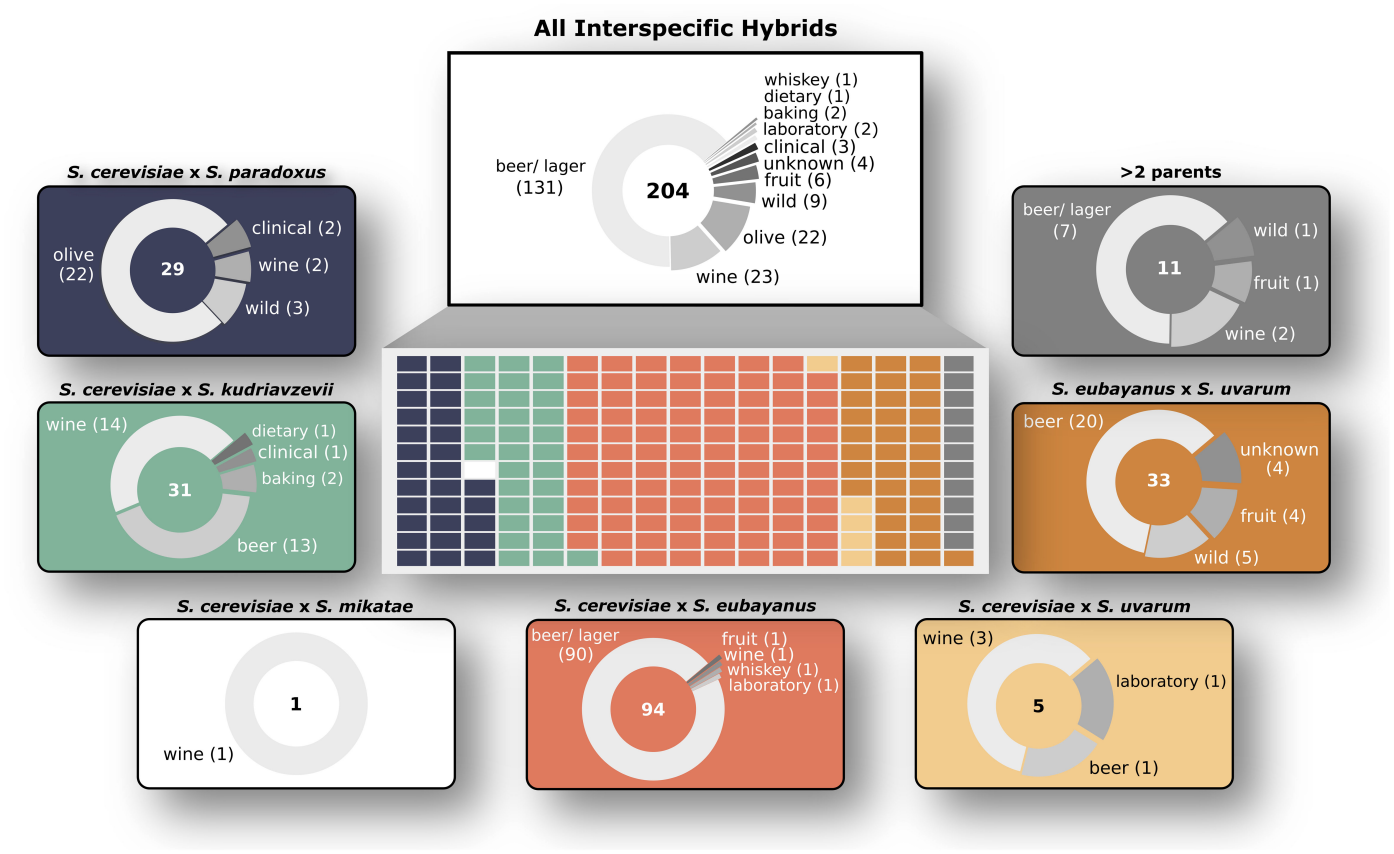
Overview of *Saccharomyces* interspecific hybrid genomes. Sampling origins of the 204 interspecific hybrid strains analyzed in this study are indicated in the top white box. Strains are divided by parent species ancestries in the central rectangle with each smaller rectangle representing a single hybrid genome, colored according to its ancestry. The seven different hybrid crosses are shown in outer boxes with sampling origins as pie charts. The total number of genomes for each cross is shown in the center of each pie chart.

Due to ongoing advances in sequencing technologies and an increasing appreciation for the industrial and evolutionary potential of yeast hybrids, a large number of hybrid genomes have been recently sequenced. Here, we employed cutting-edge bioinformatic tools and pipelines (sppIDer, AAF, MuLoYDH, BWA, FreeBayes, Control-FREEC) and custom scripts to analyze the genomic architecture of all 204 interspecific *Saccharomyces* hybrids published to date (Figure 1). This collection contains hybrids isolated from fermentation environments (beer, lager, wine, and whiskey), but also from semi-wild, human-associated environments (olives, fruit, forest soils, and clinical and laboratory settings) (Supplementary Data Sheet). Most hybrids have two parental species, but some genomes contain contributions from three or even four different species. In most cases, but not all, the species identity of the parents is known. Our analysis includes early generation hybrids with largely equal genomic content from both parent species, but the large majority of hybrids are older and have more complex evolutionary histories, i.e., they are the result of postzygotic genomic processes including LOH, recurrent miss-segregation of chromosomes in meiosis and mitosis, and repeated backcrossing with one of the parent species. Although the hybrids in this study are not the direct F1 or F2 offspring of interspecific crosses, they are derived from this “true” hybrid offspring, resulting in genetically admixed hybrid segregants and are referred to as hybrids throughout this study.

Our study provides a comprehensive analysis of the prevalence of aneuploidy, similarities in the genomic composition, and mtDNA inheritance patterns across this diverse set of hybrid genomes. We highlight genomic patterns that apply to hybrids across all species and ecological backgrounds, but also report interesting cross- and environment-specific idiosyncrasies. Our results are an important step in understanding the factors shaping divergent hybrid genomes.

## Materials and Methods

### Hybrid Data Collection and Sequencing Data Preparation

In total 204 interspecific *Saccharomyces* hybrid strains were identified from previously published studies (Almeida et al., 2014; Strope et al., 2015; Van den Broek et al., 2015; Barbosa et al., 2016; Borneman et al., 2016; Okuno et al., 2016; Zhu et al., 2016; Smukowski Heil et al., 2017; Krogerus et al., 2018; Brouwers et al., 2019; Gallone et al., 2019; Langdon et al., 2019; Pontes et al., 2019; Salazar et al., 2019; Morard et al., 2020; Turgeon et al., 2021). Raw sequencing data were obtained from the European Nucleotide Archive (https://www.ebi.ac.uk/ena). The accession number for each sequenced strain is included in Supplementary Datafile 1. Raw sequencing reads were trimmed for quality and adapters using Trim Galore v0.6.6 using default settings (Martin, 2011; Krueger, 2015).

### Inferring Phylogenetic Relationships

To place the hybrid strains in context with the eight currently recognized *Saccharomyces* species, we constructed a phylogenetic network using an assembly and annotation free approach (AAF). Annotation free approach v20171001 (Fan et al., 2015) was used with a k-mer size of 17 nucleotides and threshold frequency of five for each k-mer to be included in the analysis. To improve visualization of the complex network of hybridization, we used the distance matrix generated by AAF to create a neighbor network using the neighbor-net algorithm implemented in SplitsTree v4.17.0 (Bryant and Moulton, 2004; Huson and Bryant, 2006). We used an EqualAngle splits transformation with Optimize Boxes Iterations = 10, Daylight Iterations = 5, Spring Embedder Iterations = 0, with weights and running Convex Hull.

To understand the phylogenetic relationships of the putative parental species, we built a consensus species tree using orthogroup inference of the eight currently recognized *Saccharomyces* species. Orthologs were identified from previously assembled and annotated *Saccharomyces* genomes (Liti et al., 2009, 2013; Scannell et al., 2011; Baker et al., 2015; Naseeb et al., 2018; Bendixsen et al., 2021). *Kluyveromyces lactis* and *Torulaspora delbrueckii* were used as outgroups in the phylogenetic analysis. We used OrthoFinder v2.5.2 (Emms and Kelly, 2015, 2019) and aligned all orthologous protein-coding genes identified in the eight *Saccharomyces* species. In total, 5,672 orthogroups were identified and gene trees were constructed for each group. The consensus species tree is inferred using STAG (Emms and Kelly, 2018) and rooted using STRIDE (Emms and Kelly, 2017).

### Species Contributions to Interspecific Hybrids

We used sppIDer (Langdon et al., 2018), a highly developed hybrid detection and analysis pipeline, to re-confirm the hybrid identities of the published strains. This allowed us to determine the relative genomic contribution of each species to a given hybrid. For sppIDer, a combination of reference genomes including a representative of each of the eight currently recognized “pure” *Saccharomyces* species was used. The representative strain from each species were previously assembled and annotated (Liti et al., 2009, 2013; Scannell et al., 2011; Baker et al., 2015; Naseeb et al., 2018; Bendixsen et al., 2021) and are as follows (*species*: strain); *S. cerevisiae*: Y55, *Saccharomyces paradoxus*: N44, *Saccharomyces mikatae*: IFO1815, *Saccharomyces jurei*: NCYC3947, *Saccharomyces kudriavzevii*: CR85, *Saccharomyces arboricola*: H6, *Saccharomyces eubayanus*: FM1318, *Saccharomyces uvarum*: CBS7001. sppIDer uses BWA-MEM (Li, 2013) to map sequencing reads to the combination reference genome. The BWA output is then sorted using SAMtools (Li et al., 2009) to keep only reads that are mapped with a MQ > 3. Bedtools genomeCoverageBed is then used to determine the number of reads that are mapped to each base pair (Quinlan and Hall, 2010). To validate the efficacy of using sppIDer to detect species contributions, we tested sequencing reads from each of the pure species and found high specificity. The representative strains chosen for each species are unlikely to be the parents of the hybrids studied. Therefore, to limit any potential issues with poor mapping to the chosen representative genomes, we limited the remaining analyses to two-parent hybrids, where >85% of sequencing reads successfully mapped with high quality. The figures were created using custom Python scripts, matplotlib (Hunter, 2007) and seaborn (Waskom, 2021).

Similarly, we used mitoSppIDer (Langdon et al., 2018) to determine the relative contribution of each species to the mitochondrial genome in each hybrid strain. This analysis was done similarly to the whole genome analysis using sppIDer, however, only the mitochondrial genomes from the eight representative strains were included in the combined reference genome. Due to the significant divergence of mtDNA within the *S. eubanayus* species (Peris et al., 2014; Baker et al., 2015), two representative mitochondrial genomes from divergent clades were used. The representative mitochondrial genome from the holarctic clade was strain CDFM21L.1 and the mitochondria from the Patagonia B clade was strain FM1318. Using CDFM21L.1 Illumina reads SRR1507225 (Bing et al., 2014), the mtDNA was assembled with SPAdes (Peris et al., unpublished), implemented in the iWGS v1.1 wrapper (Zhou et al., 2016). When the *S. eubayanus* mitochondria were present within a hybrid genome, the reads mapped predominantly to only one of the two mitochondrial assemblies and thus this number was used for further analyses. We then correlated the amount of nuclear genome and mitochondrial genome inheritance both measured as the percentage of reads mapping to the dominant species. Lastly, we determined if there was a significant relationship between the percentage of nuclear genome inheritance and whether or not mitochondrial genomes were inherited from that species using a Welch's *t*-test which allows for unequal variance.

### Sequencing Read Depth Analysis

As a metric of the gain or loss of chromosomes (aneuploidy) we analyzed the depth of sequencing reads mapped to the putative parental species using sppIDer (Langdon et al., 2018). For this analysis we only included the hybrid genomes with two putative parents and excluded datasets which had >15% of sequencing reads that did not map to any of the eight parental species. This resulted in 170 hybrid genome datasets for analysis. Mean read depth was calculated for windows of ~10 kbp. Windows which had significantly high mean read depths [>2x mean read depth (log2) of the chromosome] were suspected of being repetitive elements that were poorly resolved in the genome assemblies and were therefore excluded from chromosomal read depth calculations. We determined the mean chromosome read depth and genome-wide read depth for each parental species. We also determined the total mean read depth for each chromosome (species 1 mean chromosome read depth + species 2 mean chromosome read depth), as well as the genome-wide read depth. We then determined the level of aneuploidy defined as chromosomes with a mean read depth 30% higher (gain) or lower (loss) than the genome-wide mean. In order to assess the level of variability within each hybrid genome, we determined the chromosomal variance (standard deviation squared) of mean chromosome read depth. We then determined the mean read depth change, defined as the absolute difference in read depth for each chromosome from the genome-wide read depth mean. We examined the relationship between aneuploidy (measured as chromosomal read depth) and chromosome size using linear regression for all hybrids and for each hybrid cross. The figures were created using custom Python scripts, matplotlib (Hunter, 2007) and seaborn (Waskom, 2021). Python scripts and data used for analyses are available on GitLab (https://gitlab.com/devinbendixsen/yeast-hybrid-genomic-instability).

### Assessing Patterns of Loss of Heterozygosity

We used the MuLoYDH pipeline (Tattini et al., 2019) to assess patterns of LOH. Of all genomes, only a subset of *S. cerevisiae* × *S. paradoxus* hybrids was suitable to be used in this pipeline with specifically annotated centromeric and subtelomeric regions. Furthermore, we have limited the analysis to five hybrid genomes within this cross that inherited at least 20% of the nuclear genome from each parental species. We define heterozygosity as differences in the homeologous segments of the parental subgenomes. Here we briefly describe the flow of the MuLoYDH pipeline. MuLoYDH uses BWA-MEM (Li, 2013) to map sequencing reads to each parental genome individually and to a combined genome competitively. Parental genomes used for this analysis were SK1 (*S. cerevisiae*) and N44 (*S. paradoxus*). Single-nucleotide markers were identified using the NUCmer algorithm (Kurtz et al., 2004) and are used to map LOH segments. We used the collinear mode, which determines markers chromosome-by-chromosome. Markers are then called and genotyped using SAMtools (mpileup) and BCFtools. Markers are then quality-filtered. *De novo* SNVs and indels are called using two approaches: (1) using SAMtools (mpileup) and BCFtools and (2) FreeBayes (Li, 2011; Garrison and Marth, 2012). Variants that were identified in both were kept and filtered for quality and subtelomeric regions are masked. CNVs are determined using Control-FREEC (Boeva et al., 2012) using standard mappings against both *S. cerevisiae* and *S. paradoxus* genomes. RC data was then normalized by GC-content and mappability calculated using GEM-mappability (Derrien et al., 2012). BAF-values were then calculated and LOH regions were determined and annotated.

## Results

### Genetic Ancestry of Interspecific Hybrids

**Figure 2:**
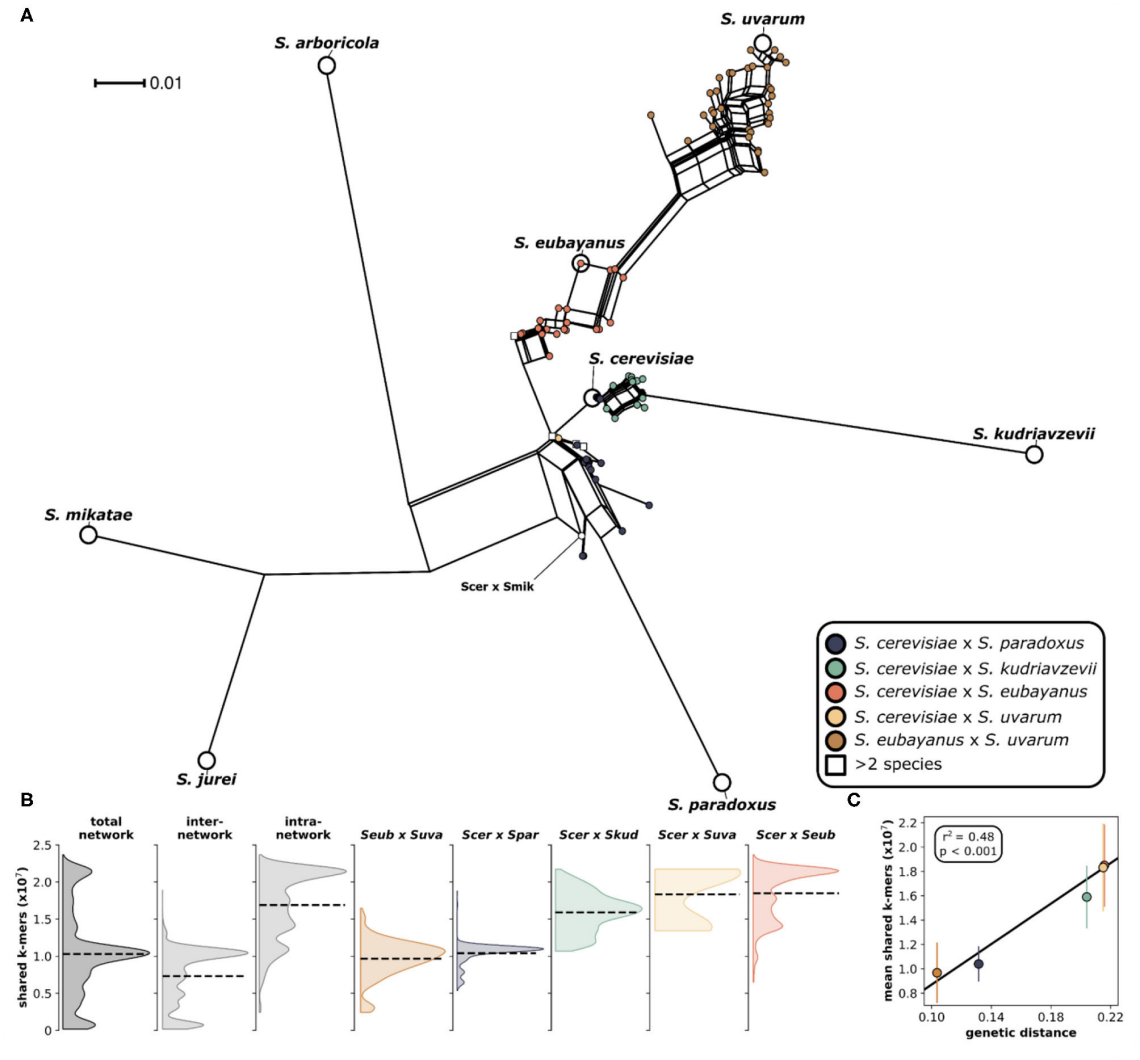
Phylogenetic network of *Saccharomyces* interspecific hybrids. **(A)** A neighbor net splits tree based on the distance matrix generated from a k-mer analysis using an assembly and alignment free approach (AAF). The analysis was performed on the trimmed sequencing reads with k-mer size of 17 and a threshold frequency of 5 for each k-mer to be retained in the analysis. Pure species are indicated with larger white circles and are labeled. Each circle indicates a hybrid and is colored according to the legend. Hybrids with more than two parents are indicated with a square. The single *S. cerevisiae* × *S. mikatae* hybrid is labeled (Scer × Smik). **(B)** Distributions of shared k-mers for each hybrid cross. Total network includes all pairwise k-mer comparisons both within and between hybrid crosses. Inter-network includes only k-mer comparisons between hybrid crosses. Intra-network includes all k-mer comparisons within each hybrid cross. Distributions for individual hybrid crosses include only k-mer comparisons with other genomes within the same hybrid cross. Dashed lines indicate the distribution mean. **(C)** Correlation between mean shared k-mers and genetic distance between the two parental species. Vertical lines indicate the standard deviation of the mean.

We analyzed 204 interspecific *Saccharomyces* hybrids to determine the relative genomic contributions from parental species to each hybrid genome. The analyzed hybrids came from six different two-parent crosses, as well as several hybrids with more than two parents (Figure 1). The majority (~76%) of these sequenced hybrids were isolated from fermentation environments, with ~64% from beer/ lager (131) and ~11% from wine (23). The remaining hybrids were isolated from various environments including olives (22), wild (9), and fruit (6). Given that the majority of sequenced hybrids were isolated from fermentation environments, the most common hybrid cross was *S. cerevisiae* × *S. eubayanus*, which is widely used in the production of beer and lager. Other hybrid crosses had significantly less sequenced hybrids to analyze, such as *S. cerevisiae* × *S. uvarum* (5) and *S. cerevisiae* × *S. mikatae* (1). Due to the significant use and vast diversity of *S. cerevisiae*, the majority (~84%) of sequenced hybrids are derived from this species. We found that building a phylogenetic network based on shared k-mers within each hybrid genome revealed that, as expected, genomes from the same hybrid cross clustered together (Figure 2A). However, the degree of genomic diversity (number of shared k-mers) and variability (their distribution around the mean) varied between crosses (Figure 2B). Despite *S. cerevisiae* × *S. eubayanus* hybrids representing the largest portion of hybrid genomes analyzed here (~46%), they tightly grouped together resulting in a distribution with a main peak at a high level of shared k-mers, indicating high genetic similarity within this cross. Likewise. *S. cerevisiae* × *S. uvarum* hybrids showed high genetic similarity and low diversity. *S. cerevisiae* × *S. paradoxus* and *S. eubayanus* × *S. uvarum* hybrids had the lowest amount of shared k-mers within each hybrid cross. This pattern revealed a significant correlation between the mean shared number of k-mers within a hybrid cross and the genetic distance between the two putative parental species (Figure 2C). The more distant the parental species were genetically, the more similar were the genomes of the sampled hybrids. To account for varying levels of read depth between hybrid genomes, we also repeated the k-mer analysis with a lower cut-off for inclusion (*n* = 2) and found the same pattern suggesting that it is not directly linked to read depth (Supplementary Figure 1).

**Figure 3:**
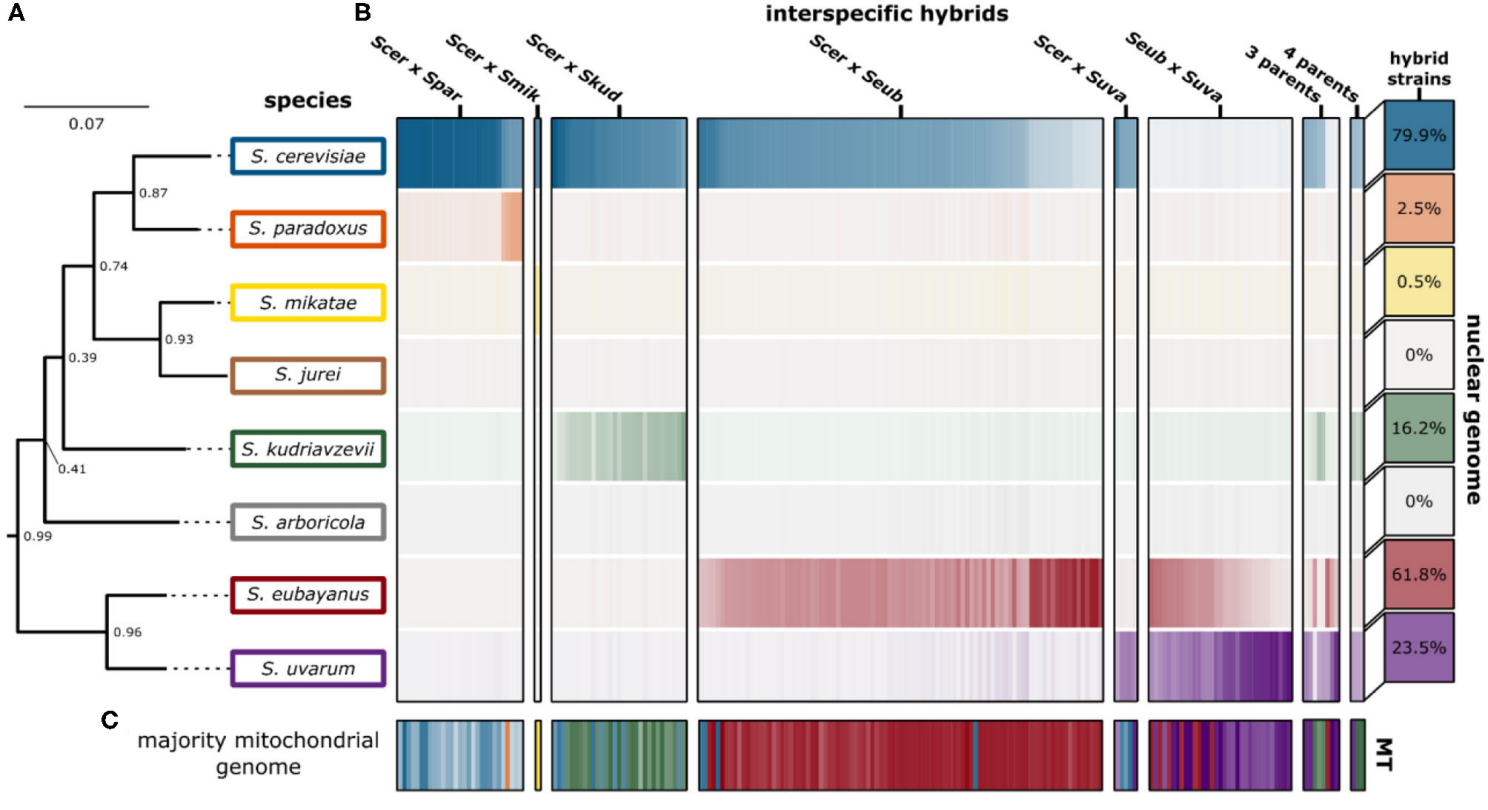
Genetic ancestry of interspecific hybrids. **(A)** The phylogenetic tree indicates the phylogenetic relationship of the eight *Saccharomyces* species. The support values indicate the proportion of times that the bipartition is seen in each of the individual species tree estimates. Branch lengths represent the average number of substitutions per site across the sampled gene families. **(B)** Genomic contributions are calculated as the proportion of reads mapping to a representative of each species. The 204 interspecific hybrid strains span the horizontal axis, with the genomic contribution of each hybrid strain indicated. The intensity of each color indicates the genomic contribution for each species, with low (white) and high intensity indicating low and high genomic contributions, respectively. The percentage of hybrid strains in this study (*n* = 204) with significant genomic contributions from each *Saccharomyces* species is indicated in boxes on the right. **(C)** The majority mitochondrial genome within each hybrid strain is shown with similar coloring. Mitochondrial genome contributions from each species are shown in Supplementary Figure 4.

We were able to recapitulate and confirm the previously reported genetic ancestry (species contributions) for most of the interspecific hybrids collected from published studies (Figure 3). For some, in particular the “relic” hybrids generated from *S. cerevisiae* × *S. paradoxus*, smaller introgressions were detected, however the contribution was not significant across the whole genome (Supplementary Figure 2). We found that for most hybrid crosses, the collected hybrid genomes represent a range of genetic contributions from each species (Supplementary Figure 3). For the remaining analyses we focused on characterizing the 170 hybrid genomes with only two parental species and which had >85% of sequencing reads mapping with high quality.

### Mitochondrial Genome Inheritance Patterns

**Figure 4:**
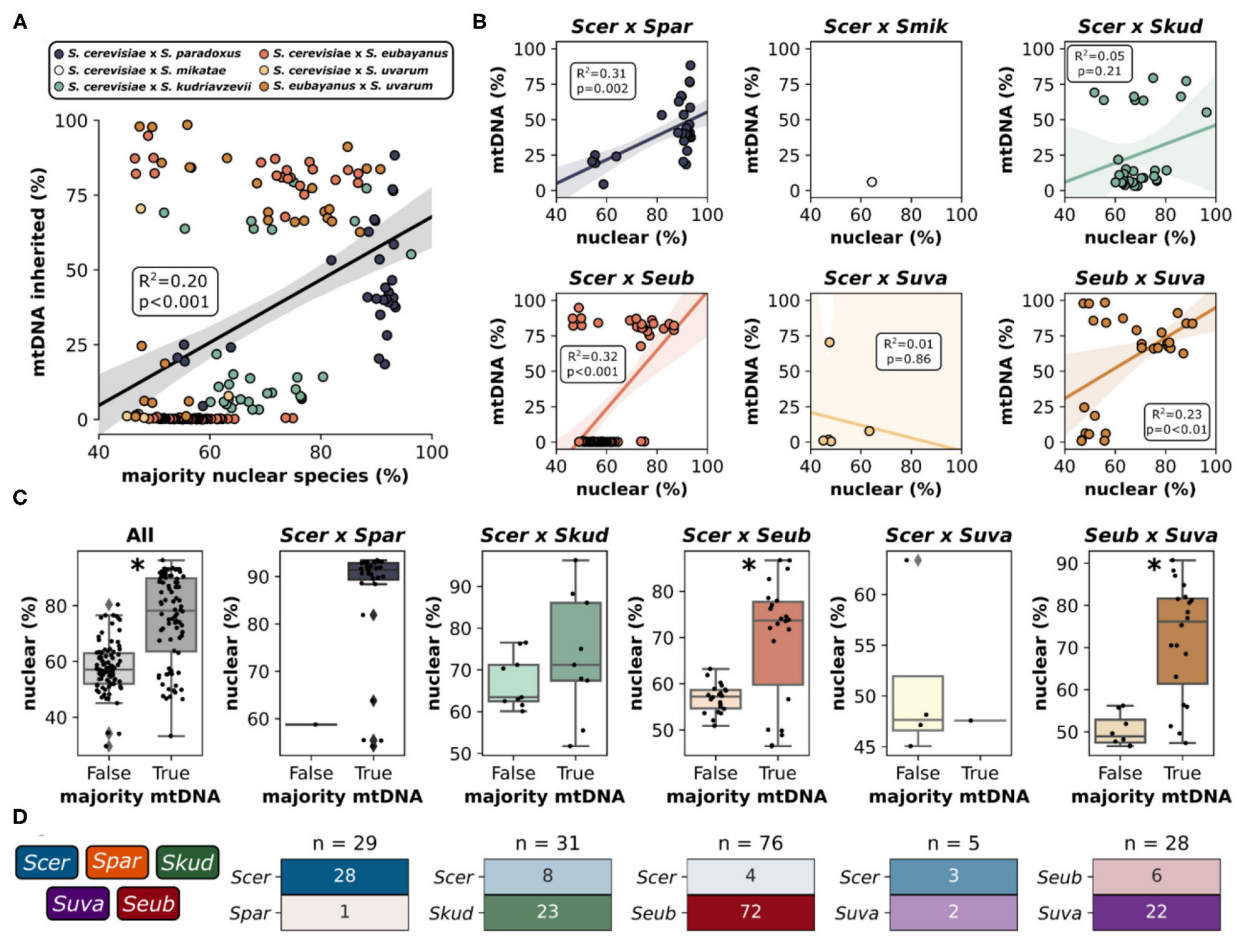
Mitochondrial inheritance patterns in interspecific hybrids. **(A)** Relationship between the percentage (%) of reads mapping to the majority nuclear species and mtDNA inherited from the majority nuclear species for all hybrid crosses. Data points are colored according to the hybrid cross. Linear regression is shown as black line with 95% confidence interval. X-axis extends below 50% as some of the Scer × Seub hybrid genomes remained unmapped or mapped to a third parent species **(B)** Relationship between the majority nuclear species and mtDNA inheritance for each pairwise hybrid cross. **(C)** Mitochondrial inheritance as a function of nuclear species majority (% reads mapping to that species). False indicates that the most frequent mitochondrial genome is not from the majority nuclear genome. True indicates that the most frequent mitochondrial genome and the majority nuclear genome are from the same species. Asterisk indicates a significant difference (*p* < 0.05) from Welch's *t*-test. Scer × Spar and Scer × Suva could not be tested as one of the categories only had a single value. **(D)** Mitochondrial inheritance for each species within each hybrid cross. *n* indicates the number of hybrids within a particular hybrid cross. The heatmap is colored and labeled according to the percentage of each hybrid cross that dominantly inherited mitochondria from each species. Colors and color intensity match the color scheme in Figure 3.

Mitochondria in yeast are inherited from both parents during a hybridization event, however, one of the mitochondrial genomes is retained while the other is rapidly lost resulting in homoplasmic (either uniparental or recombinant) offspring. Accordingly, for most hybrid genomes within this study, sequencing reads mapped predominantly to the mitochondrial genome from a single parent with minor introgressions from other species (Figure 3C, Supplementary Figure 4). Generally, the relative contribution of mitochondrial DNA inherited from a species was significantly correlated with the relative contribution of nuclear DNA inherited from that species (Figure 4A). This trend was however not universally retained when each hybrid cross was studied independently (Figure 4B). Most notably, the mtDNA of *S. cerevisiae* × *S. kudriavzevii* hybrids (*n* = 31) is not predominantly inherited from the majority nuclear parent. Other exceptions to this rule may be better explained by low sample size (*S. cerevisiae* × *S. uvarum*). The amount of nuclear DNA inherited from the majority species reliably predicted whether or not the majority of the mitochondrial genome was also inherited from that same species (Figure 4C). Once again, the relationship was not significant in *S. cerevisiae* × *S. kudriavzevii* hybrids.

In some hybrid crosses, mitochondrial genomes were predominantly inherited from a single species (Figure 4D). In *S. cerevisiae* × *S. paradoxus* hybrids, 97% of hybrids inherited mitochondrial genomes from *S. cerevisiae*. This is not surprising, given that *S. cerevisiae* is the majority nuclear species in most of this hybrid cross, with minor introgressions from *S. paradoxus*. However, in other hybrid crosses, where species contributions are more dispersed, patterns of preference still exist. In *S. cerevisiae* × *S. kudriavzevii* hybrids, the majority (74%) inherited the *S. kudriavzevii* mitochondrial genome. Similarly, *S. uvarum* mitochondria were inherited in 79% of *S. eubayanus* × *S. uvarum* hybrids. Most notable however is that in *S. cerevisiae* × *S. eubayanus* hybrids, mitochondrial genomes from *S. eubayanus* were almost exclusively inherited (~95%), despite nuclear genome contributions from *S. eubayanus* ranging from ~25 to ~80% (Supplementary Figure 3B). These results are in agreement with previous studies suggesting that the hybrids benefit from the cold tolerance of the non-*cerevisiae* parent in these crosses (Baker et al., 2019; Li et al., 2019). Due to the large divergence of mitochondrial genomes within *S. eubayanus*, we mapped reads to the mitochondrial genomes from two different clades (Holarctic and Patagonia B). We found that hybrids with *S. eubayanus* mitochondrial genomes mapped almost exclusively to the holarctic reference genome (Supplementary Figure 5).

### Aneuploidy Is Prevalent in Interspecific Hybrids

**Figure 5:**
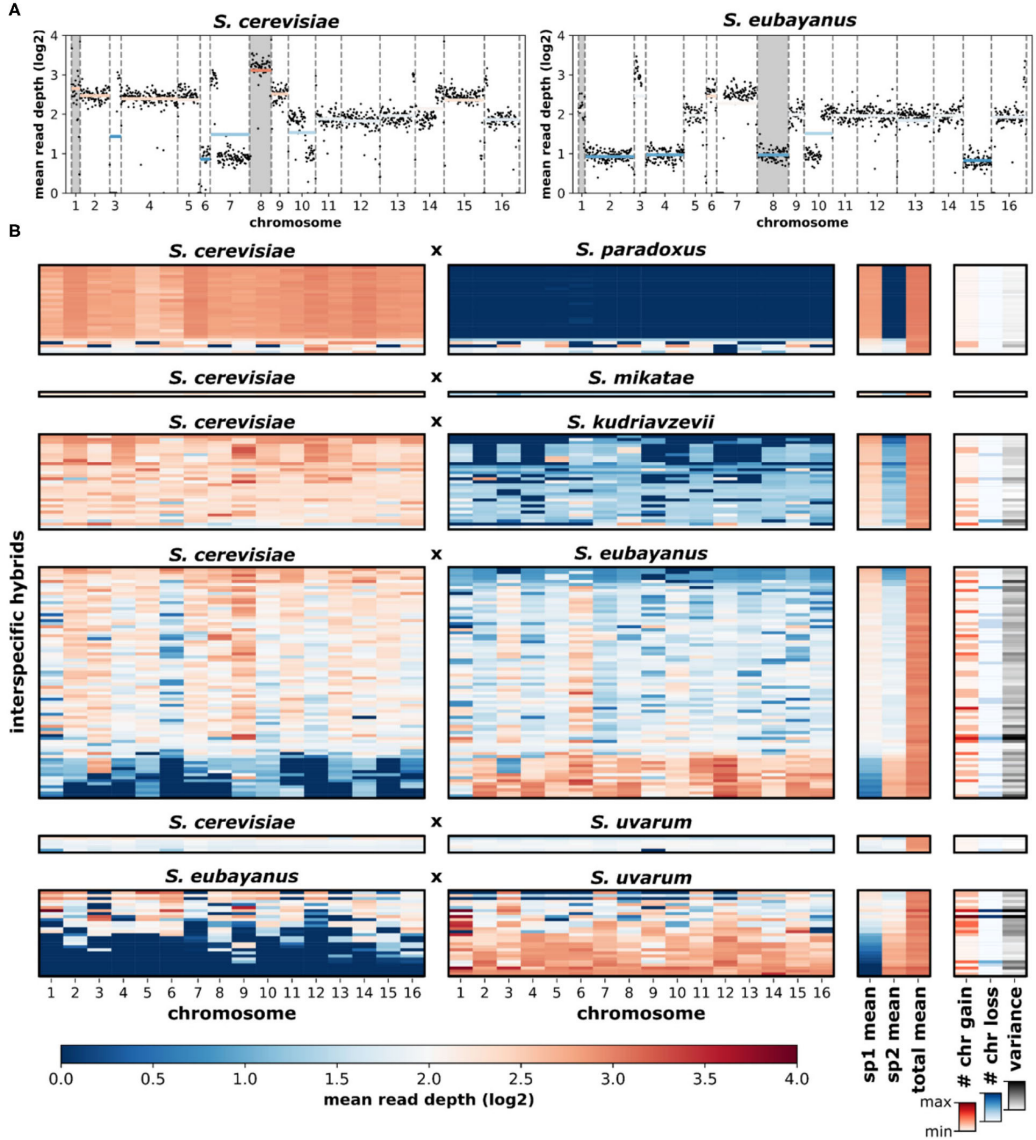
Sequencing read depth of *Saccharomyces* interspecific hybrids. **(A)** An example of a hybrid (CBS1483) with reads mapped to the putative parental species, *S. cerevisiae* and *S. eubayanus*. Black dots indicate the read depth of ~10 kbp windows. Dashed vertical lines separate chromosomes. Horizontal colored lines indicate mean chromosomal read depth. Chromosomes with elevated read depths (aneuploidy) are indicated with gray backgrounds. **(B)** Mean chromosomal read depths of 170 hybrid genomes. Rows are individual hybrid genomes. Genomes are presented in the same order as Figure 3 and are grouped by hybrid cross. Genome-wide read depth means for each parental species (sp1, sp2) and the total (sp1+sp2) genome-wide mean are shown. The number of high (# chr gain) and low (# chr loss) read depth chromosomes were defined as chromosomes with a mean read depth 30% higher or lower than the genome-wide mean. Chromosomal read-depth variance was calculated as the variance (s^2^) of the genome-wide mean.

**Figure 6:**
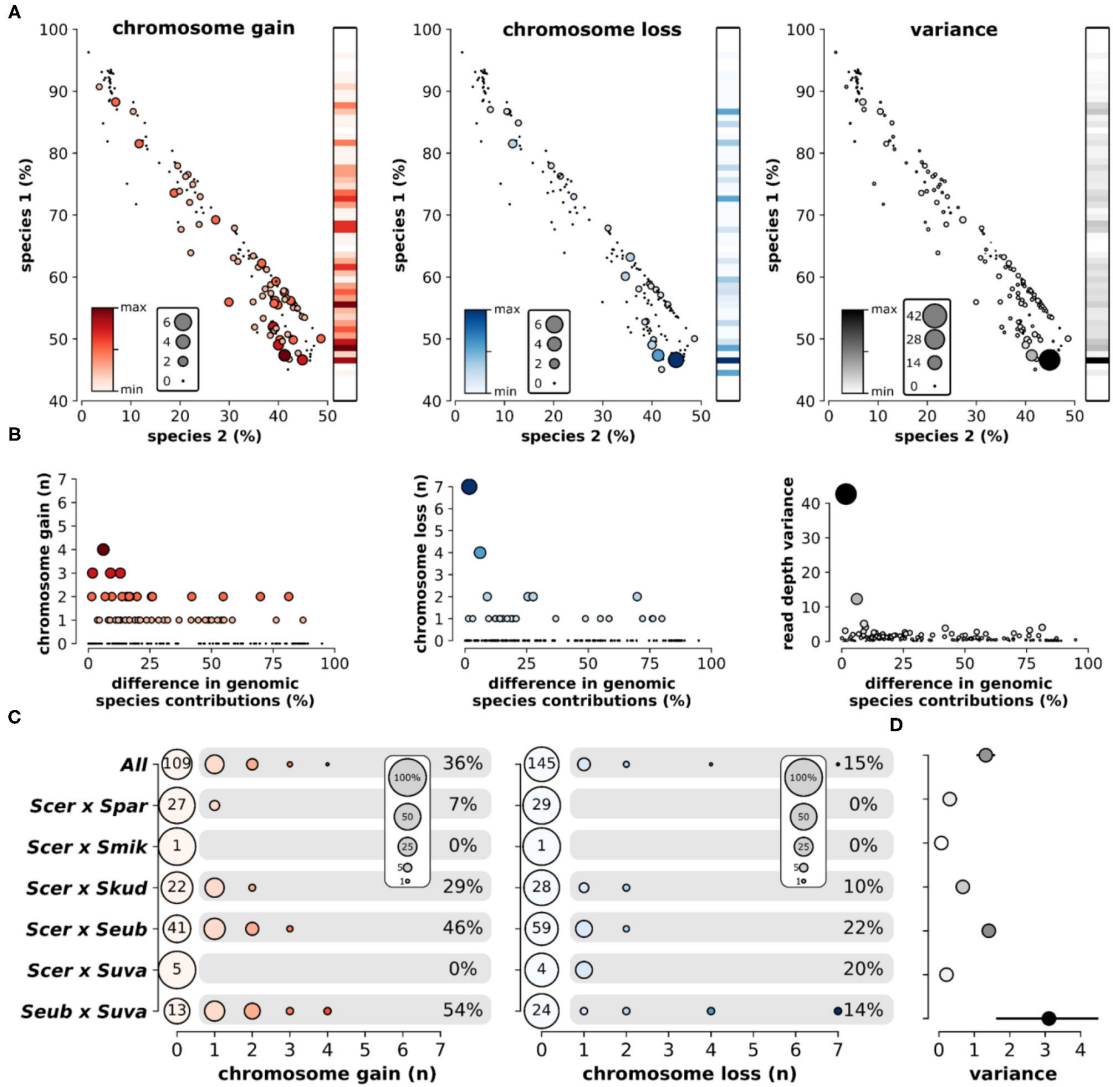
Aneuploidy and sequencing read depth variance among hybrid crosses. **(A)** The relationship of percent reads mapping to species 1 and species 2 and the number of chromosomes gained (red), chromosomes lost (blue), and read depth variance (black). Color and size of nodes indicate the number of chromosomes or read depth variance. Heatmaps indicate the mean level gain, loss, or variance for each level of species 1 inheritance. **(B)** Relationship between the difference in genomic contributions from each species (sp1-sp2) and aneuploidy. Color and size of nodes indicate the number of chromosomes or read depth variance. **(C)** The number of chromosomes gained or lost in each hybrid cross. Node size indicates the percentage of genomes within each hybrid cross. Numbers at point zero indicate the number of genomes without a gain or loss. Percentages indicate the proportion of genomes within that hybrid cross with at least one chromosome gain or loss. **(D)** The mean and standard error of chromosome read depth variance for each hybrid cross.

Overall, the loss and gain of chromosomes (aneuploidy) was common in interspecific hybrids. Using variation in chromosomal sequencing read depths as a metric of aneuploidy (Figure 5A), we found that ~44% of all hybrid genomes contained deviations in read depth (>30% of the genome-wide mean) for at least one chromosome (Figure 5B). Elevated read depth (# chr gain) was observed in ~36% of the hybrids. Only ~15% showed significant decreases in read depth (# chr loss). Interestingly, ~6% of hybrids had both significant gain and significant loss of chromosomal read depths within the same genome. Analyzing sequencing data of the parental species revealed no such deviations in chromosomal read depth (Supplementary Figure 6). Sequencing read depth deviations in hybrids resulted in a range of chromosome level read depth variances, defined as the variance in mean read depth of the 16 chromosomes within a hybrid genome. The range of chromosome gains, losses and overall variance fluctuate across the scope of genetic ancestry and species contributions (Figure 6A). The most notable trend is that chromosome gains and losses and the resulting variance are often minimal when the majority of the hybrid genome maps to a single parental species (Figure 6B). However, as the percentage of reads mapping to the two majority parental species approached equal contributions (~50%) the variance often increased. The chromosome gains and losses were not equally distributed among all hybrid crosses (Figure 6C). In some crosses, such as *S. cerevisiae* × *S. paradoxus*, chromosome gains were rare (~7%) and losses did not occur. However, in other hybrid crosses, aneuploidy was especially prevalent. In *S. eubayanus* × *S. uvarum* hybrids, gains were found in ~54% of genomes, with ~14% having chromosome losses. Similarly, *S. cerevisiae* × *S. eubayanus* hybrids also had elevated amounts of chromosome gains and losses (~46 and 22%, respectively). These variations in chromosome gains and losses resulted in notable variations in mean chromosome read depth variance for each hybrid cross (Figure 6D). Interestingly, this trend did not correlate with genetic distance between the two parental species.

**Figure 7:**
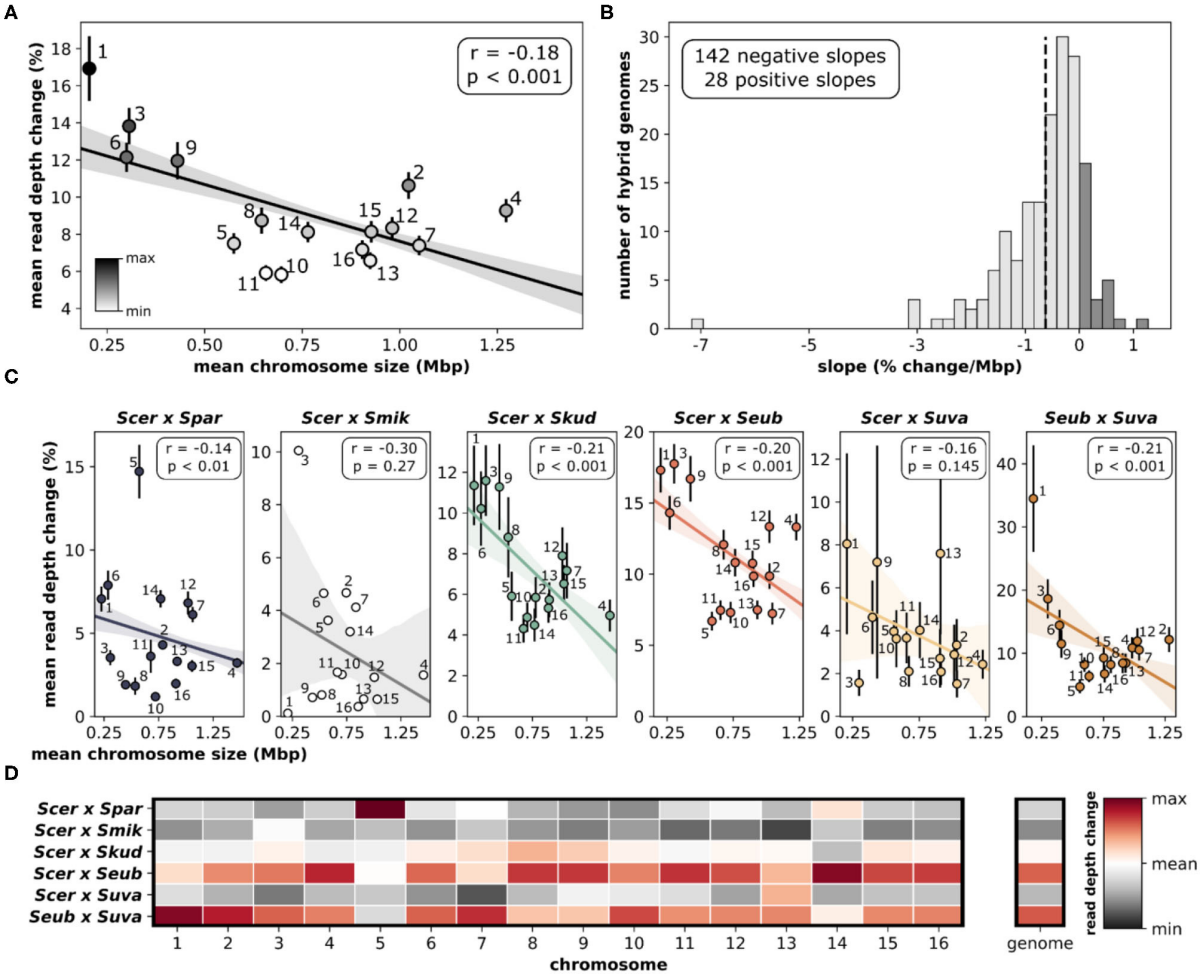
Relationship between chromosome size and aneuploidy. **(A)** Linear correlation between chromosome size and delta mean read depth. Delta mean read depth is calculated as the absolute difference between the total chromosomal read depth (species 1 + species 2) and the total genome read depth. Means and standard error for each independent chromosome are shown and labeled. The linear regression line with 95% confidence intervals is shown. **(B)** The distribution of slopes generated from linear regressions for each hybrid genome (*n* = 170). All of the linear regressions for each hybrid cross are depicted in Supplementary Figure 7. Dashed vertical line indicates the mean. **(C)** Linear correlations between chromosome size and delta mean read depth for each hybrid cross. **(D)** Mean read depth change for chromosomes compared across hybrid crosses. Each chromosome (column) has been normalized for comparisons. Red indicates the maximum read depth change for that chromosome, white indicates the overall mean and dark gray indicates the minimum. Mean read depth change for the whole genome is also normalized and colored.

Assessing the change in sequencing read depth (aneuploidy) for each individual chromosome within a hybrid genome revealed significant patterns. Quantifying the mean read depth change, which incorporates both gains and losses, showed that smaller chromosomes were more likely to have variations in copy number (Figure 7A), whereas larger chromosomes were more likely to have lower amounts of variation. The relationship between chromosome size and aneuploidy was significant (*r* = −0.18, *p* < 0.001). When each hybrid genome was analyzed individually, the vast majority (~84%) had negative slopes when correlating change in read depth and chromosome size (Figure 7B, Supplementary Figure 7). This suggests a similar trend in individual hybrid genomes, where smaller chromosomes are more variable than larger chromosomes. The significant relationship between chromosome size and aneuploidy is seen in most hybrid crosses (Figure 7C). The two hybrid crosses that failed to reach significance (*S. cerevisiae* × *S. mikatae, S. cerevisiae* × *S. uvarum*) had small sample sizes (*n* = 1 and 5, respectively). Although the relationships between chromosome size and aneuploidy are maintained between hybrid crosses, there are differences in which chromosomes are variable (Figure 7D). Overall, *S. cerevisiae* × *S. eubayanus* and *S. eubayanus* × *S. uvarum* hybrids were the most variable. A notable deviation from this pattern is chromosome 5 in *S. cerevisiae* × *S. paradoxus* hybrids.

### Patterns of Loss of Heterozygosity in Interspecific Hybrids

**Figure 8:**
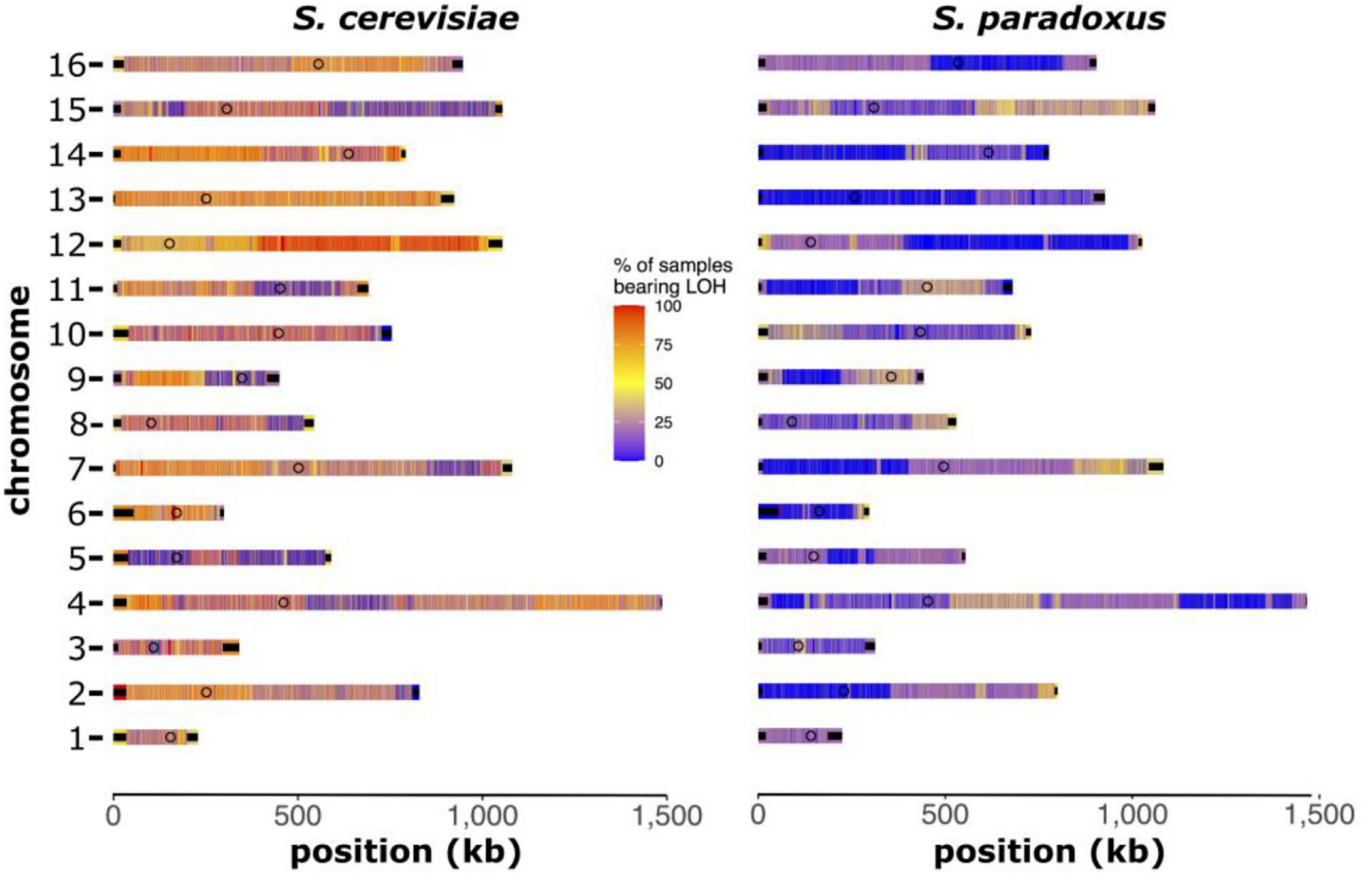
Chromosomal maps of loss of heterozygosity (LOH). Heatmaps of each chromosome depicting the regions with high (red) or low (blue) levels of LOH. Regions of high LOH are homozygous for markers from that parental reference genome. Heatmap color indicates the percentage of samples bearing LOH at that region. This analysis was performed on *S. cerevisiae* × *S. paradoxus* hybrids with at least 20% of the hybrid genome inherited from each species (*n* = 5).

Using a subset of *S. cerevisiae* × *S. paradoxus* hybrids that inherited at least 20% of the nuclear genome from each parental species (five genomes), we explored patterns and trends in LOH. We identified regions within each chromosome that maintained a higher propensity of LOH suggesting that they harbor homozygous markers from either of the parental species (Figure 8). Overall, hybrids were more likely to have homozygous markers from *S. cerevisiae*, which agrees with the larger proportion of these hybrids mapping to that species (54–64%). Across the hybrids analyzed, we found that ~30% of the hybrid genomes were identified as regions with LOH. Using the chromosome maps, we identified regions that had higher levels of LOH. Most notably, large portions of chromosome 12 were almost exclusively homozygous for markers found in *S. cerevisiae*. Interestingly, chromosome 5 showed limited amounts of homozygosity for either *S. cerevisiae* or *S. paradoxus*, indicating a high level of heterozygosity. None of the genomes included in this analysis showed signs of aneuploidy (based on read-depth analysis), suggesting that that these LOH patterns were not caused by chromosome losses (hemizygosity).

## Discussion

Hybridization between divergent species generates novel genomic combinations and offers insights into the genomic architecture underlying fitness. Interspecific hybridization has been shown to have severe genomic consequences (Smukowski Heil et al., 2017; Zhang et al., 2020), often resulting in highly unstable genomes characterized by instability (Dunn et al., 2013; D'Angiolo et al., 2020; Morard et al., 2020; Steensels et al., 2021). Leveraging high-throughput sequencing data from 204 interspecific hybrids within the *Saccharomyces* genus, we offer a novel perspective on patterns of genetic ancestry, mtDNA inheritance, aneuploidy and LOH. We tailored and integrated existing bioinformatic pipelines (such as sppIDer and MuLoYDH) with other bioinformatic tools (such as AAF) and custom Python scripts, offering the unique opportunity to expand our knowledge of hybrid genomic architecture and facilitate the comparison of the genomic ancestry of hybrids with diverse genetic backgrounds.

The hybrid genomes analyzed here are the result of vastly divergent crosses, made from parent yeast species as genetically distant as humans and mice (Shen et al., 2018). For this study, we were constrained by the high-throughput sequencing datasets available for interspecific hybrids (Figure 1). The data is not uniformly distributed among the possible hybrid crosses within *Saccharomyces*. We recognize that this can limit and potentially bias interpretations across genetic ancestries due to varying sample sizes. Here, we have focused on general patterns and therefore hope to limit speculation in crosses with limited available data. Interestingly, we found that as genetic distance increased (from ~10 to ~22% nucleotide differences genome-wide), the number of shared sequences in hybrid genomes also increased (Figure 2C). Thus, more genetically divergent parents resulted in genomically more similar hybrids. The reasons for this are yet unclear but this might be the result of stronger negative epistasis at larger genomic distances constraining the number of viable genomic combinations in hybrids. This is interesting with respect to the evolution of reproductive isolation in this species group. Recent work suggests that a large contributor to inviability and sterility in homoploid yeast hybrids is the failure of homeologeous chromosomes to pair and antirecombination, causing chromosomal missegregation during meiosis (Ono and Greig, 2019; Bozdag et al., 2021). When antirecombination was suppressed in *S. cerevisiae* × *S. paradoxus* hybrids, viability of hybrid gametes increased 70-fold (Bozdag et al., 2021). Another process that can lead to increased rates of aneuploidy upon hybridization is the gradual loss of chromosomes through both meiosis and mitosis (Sipiczki, 2018, 2019). The remaining contributions to reproductive isolation between *Saccharomyces* yeast species are likely combinations of Dobzhansky-Muller incompatibilities (DMIs), i.e., complex negative epistatic interactions involving multiple loci with weak fitness effects, which are difficult to detect statistically (Li et al., 2013) and have so far only been conclusively demonstrated to cause mortality in a large set of intraspecific S. cerevisiae crosses (Hou et al., 2014). The patterns found here, of increased genomic similarity in more divergent crosses, are consistent with complex DMIs restricting hybrid genomic architecture.

Aneuploidy, the loss or gain of a chromosome, is a common result of hybridization. In *S. cerevisiae* × *S. paradoxus* hybrids, chromosomal non-disjunction leaves a substantial portion of F1 spores (the recombined gametic products of hybrid meiosis) with at least one aneuploidy (Boynton et al., 2018; Rogers et al., 2018). Here, we found that across all hybrid genomes, aneuploidy was very frequent with 44% of genomes having at least one significant deviation in sequencing read depth. The majority of these aneuploidies were chromosomal gains (36%), fewer were chromosome losses (15%). In a recent study on 237 laboratory *S. cerevisiae* × *S. paradoxus* F2 hybrids, we found that 88% of all genomes contained aneuploidies (Zhang et al., 2020). This supports the notion that early hybrid generations contain more aberrant chromosome numbers than later generation hybrids that are more stabilized, as is the case for many of the genomes included in this study. This result also shows that the prevalence of aneuploidy is more than twice as high in hybrid meiosis outcomes compared to regular meiosis within *S. cerevisiae*, where aneuploidy was recently reported to affect 21% of all crosses, with variation between clades (Scopel et al., 2021).

We found that aneuploidy prevalence strongly depended on the genetic background of the hybrid cross. Among cross types, aneuploidy ranged from none of the genomes containing aneuploidies to 54% of the genomes having aneuploidies (Figures 6C,D). Against our expectation, there was no relationship between aneuploidy and genetic distance between parental species. But interestingly, aneuploidy was most frequent when genomic contributions from the two parental species approached equal shares in hybrid genomes (Figures 6A,B). When hybrid genomes harbored genomic content mostly from a single species, with only minor introgressions from the other species, read depth variance was consistently low. Equal genomic contributions are indicative of the hybrid's evolutionary history, suggesting the hybridization event was relatively recent, with limited time for stabilizing postzygotic processes and backcrossing. Older hybrids with a more complex history likely benefit from genome stabilization, allowing them to return to euploidy, which is reflected in our data.

Another interesting pattern is that aneuploidy in yeast is more common in smaller chromosomes (Kumaran et al., 2013; Zhu et al., 2016; Peter et al., 2018; Gilchrist and Stelkens, 2019). We found the same relationship between chromosome size and aneuploidy (measured as mean read depth change) in this large set of interspecific yeast hybrids (Figure 7A). As chromosome size increased, the rate of aneuploidies decreased, likely due to the higher number of genes with important functions on larger chromosomes (Scopel et al., 2021). Smaller chromosomes tend to have fewer genes resulting in a smaller chance of dosage sensitivity. Other factors may also play a role in this, e.g., aneuploidy of the largest yeast chromosome 4 has been shown to cause a delay for entry into the cell cycle (Torres et al., 2007), likely due to the increased biological burden of protein synthesis of the extra chromosome copy. Together, this results in selection against aneuploidy of large chromosomes. In agreement with this, we found that larger chromosomes (including chromosome 4) were less likely to have gains or losses in these hybrid genomes.

Aneuploidy has been viewed as a double-edged sword with potential for adaptation (especially to stressful environments) but also fitness costs (Tsai and Nelliat, 2019). So far, aneuploidy has only been demonstrated to cause variation in fitness and adaptation in non-hybrid yeast crosses (e.g., Yona et al., 2012). For example, Scopel et al. (2021) report that whether a specific aneuploidy was neutral, detrimental or beneficial depended on the genetic background of crosses involving different strains of *S. cerevisiae*. It is reasonable to expect the same to be true in hybrids. Indeed, several industrial hybrid strains are stable aneuploids (Dunn et al., 2005; Peris et al., 2012; Okuno et al., 2016) and experimental evolution has recovered aneuploidies in different interspecific hybrids (Peris et al., 2017, 2020; Gallone et al., 2019; Langdon et al., 2019). In fact, due to the much higher frequency of chromosome missegregation in hybrid meiosis, we predict aneuploidy to feature more prominently in the adaptive evolutionary history of hybrid strains, simply because there are more occasions to recruit extra copies of genes located on aneuploid chromosomes for adaptation (Gilchrist and Stelkens, 2019).

Our results on mitochondrial DNA inheritance patterns confirm previous findings that hybrids usually retain mitochondria from the majority nuclear genome parent. For instance, it has been shown that *S. cerevisiae* × *S. kudriavzevii* hybrids with *S. kudriavzevii* mtDNA do not tolerate large losses of *S. kudriavzevii* nuclear genomic content, likely due to important species-specific interactions of proteins encoded in nuclear and mitochondrial genes (Peris et al., 2012, 2018). However, we saw remarkable exceptions to this rule, e.g., in *S. cerevisiae* × *S. eubayanus* hybrids that contained almost exclusively *S. eubayanus* mitochondria, even in genomes with a majority *S. cerevisiae* nuclear genome (Supplementary Figure 3B). These hybrids have been found in low or moderate temperature environments within the Northern European limit of the grape vine distribution, and mostly in low temperature lager fermentation environments (Figure 1), suggesting that these hybrids benefit from cold tolerance mitochondrial genes of the non-*cerevisiae* parent (Baker et al., 2019; Li et al., 2019). Only four hybrids within this cross inherited the *S. cerevisiae* mtDNA and three of these had a nuclear genome that was predominantly inherited from *S. cerevisiae* (>70%). In *S. cerevisiae* × *S. uvarum* hybrids it has been shown that mitochondrial inheritance patterns are dominated by one species and that the dominant mitochondrial parent is strain and cross specific (Verspohl et al., 2018). Therefore, mitochondria from certain *S. cerevisiae* strains were universally inherited, regardless of which *S. uvarum* strain it was crossed with. Similarly, *S. uvarum* strains were identified exhibiting dominant inheritance pattern independent of the *S. cerevisiae* mating partner. This suggests interactions between mtDNA and nuclear DNA or potentially be influenced by the mtDNA itself. Additionally, mitochondrial retention in *S. cerevisiae* × *S. uvarum* hybrids was recently shown to be heavily influenced by the effects of mitochondrial genes on nuclear expression and fitness, which in turn often depend on environmental factors (Hewitt et al., 2020). For instance, in rich media at cold temperatures, *S. uvarum* mitochondria were retained, whereas *S. cerevisiae* mitochondria were retained on non-fermentable carbon sources regardless of temperature. Thus, both intrinsic genetic (co-evolutionary constraints between mitochondrial and nuclear genes), and environment-dependent adaptive advantages may explain the patterns we observe here.

In the aftermath of faulty chromosomal segregation in yeast hybrid meiosis, various mechanisms can lead to the LOH, including the loss of entire chromosomes of one of the parental subgenomes (hemizygosity), base mismatch repair during homeologous recombination of heterozygous sites and break-induced replication (D'Angiolo et al., 2020). LOH events have recently been shown to occur at a consistently higher rate than mutations indicating a vital role in genome evolution (Dutta et al., 2021). Here, we found in a subset of ecologically diverse *S. cerevisiae* × *S. paradoxus* hybrids, that LOH was common, at approximately 30% of genomic regions. The majority of these regions were homozygous for *S. cerevisiae* genetic markers. It is interesting that these five hybrids share common LOH regions, despite being isolated from different ecological niches (olives, soil and wine). There are vast regions on chromosome 12 primarily homozygous for *S. cerevisiae* markers, whereas chromosome 5 was largely left heterozygous. Although our analysis here is quite limited in sample size (five genomes only), the patterns we see may be indicative of intrinsic genetic incompatibilities between divergent genes located in these regions. LOH has been shown to allow hybrids to overcome reproductive isolation and enables introgression between vastly divergent yeast species (D'Angiolo et al., 2020). Alternatively, LOH can be viewed as maximizing fitness potential within the hybrid genome. There is evidence for LOH to play a major role in the fitness and adaptation of yeast both in naturally occurring hybrids and interspecific crosses generated and evolved in the laboratory (Smukowski Heil et al., 2017; Lancaster et al., 2019). However, the small sample size examined here does not allow for strong conclusions about these complex interactions.

## Conclusions

In summary, our analysis provides evidence for increased similarity of hybrid genomes with increasing parental sequence divergence. This is interesting, considering that the more divergent two parental genomes are, the more can potentially go wrong during hybrid meiosis. On the other hand, more sequence differences lead to less efficient chromosome pairing in meiosis, which in turn leads to fewer meiotic divisions and less recombination and overall, potentially leading to more similar hybrid offspring. It is also important to note that all the hybrid genomes included in this study are viable outcomes of hybridization that had time to stabilize their genomes through postzygotic processes since the hybridization event. Thus, the patterns we observe may be caused by stronger negative epistasis at larger genomic divergence, putting constraints on hybridization outcomes. We also provide the first evidence for higher genome stability (less aneuploidy) the more the parental genomic contributions deviated from a 1:1 ratio in the hybrid genome. However, some questions remain. For instance, do hybrid genomes contain different levels of structural variation than their parents, such as copy number variation, translocations or inversions? Since all sequence data available for hybrid yeast genomes to date is short read data, analysis of structural variants has been limited. In addition, most genomic data so far comes from only two yeast species (*S. cerevisiae* and *S. paradoxus*). This is expected to change soon with high quality long read assemblies becoming more affordable. Another open question is about the role of transposable elements (TEs) in hybrid yeast adaptation and speciation. Hybridization has been shown to not increase the rate of TE mobilization in natural and laboratory interspecific crosses (Hénault et al., 2020; Smukowski Heil et al., 2021). But TE copy numbers in hybrids were strongly dependent on the specific genotypes used for the cross (Hénault et al., 2020) and interestingly, species-specific mitochondrial inheritance changed transposition rate by an order of magnitude (Smukowski Heil et al., 2021). Scrutinizing patterns and predictors of genomic instability in hybrids as we have done here, can potentially advance our understanding of the role of hybridization in adaptation to environmental stress, the evolution of drug resistance, and cell line disorders.

## Data Availability Statement

Publicly available datasets were analyzed in this study. The datasets analyzed for this study can be found in the European Nucleotide Archive (https://www.ebi.ac.uk/ena/browser/home). The accession numbers for each hybrid genome are shown in Supplementary Data Sheet 1.

## Author Contributions

DB and RS conceptualized and designed the study and wrote the manuscript. DB performed data analysis and data visualization. DP provided assistance with sppIDer scripts and performed mitochondrial genome assembly of the Holarctic *S. eubayanus* strain. All authors read, commented, and approved the final manuscript.

## Funding

This work was supported by the Swedish Research Council (2017-04963 to RS), the Knut and Alice Wallenberg Foundation (2017.0163 to RS), and the Wenner-Gren Foundations (UPD2018-0196, UPD2019-0110 to DB). Work performed at the National Genomics Infrastructure (NGI)/Uppsala Genome Center (project SNIC 2019/8-23) has been funded by RFI/VR and Science for Life Laboratory, Sweden. DP is a postdoctoral researcher funded by the Research Council of Norway grant no. RCN 274337 and a senior researcher supported by the Valencian International University (VIU).

## Conflict of Interest

DP declares receiving royalties from VIU based on publication productivity. The remaining authors declare that the research was conducted in the absence of any commercial or financial relationships that could be construed as a potential conflict of interest.

## Publisher's Note

All claims expressed in this article are solely those of the authors and do not necessarily represent those of their affiliated organizations, or those of the publisher, the editors and the reviewers. Any product that may be evaluated in this article, or claim that may be made by its manufacturer, is not guaranteed or endorsed by the publisher.
