# Supplementary material for: Patterns of Genomic Instability in Interspecific Yeast Hybrids With Diverse Ancestries
Source: Front Fungal Biol. 2021 Oct 12;2:742894. doi: 10.3389/ffunb.2021.742894 (PMC10512264; doi:10.3389/ffunb.2021.742894)
Supplement: Supplementary file 1 [file Data_Sheet_1.DOCX]

Supplementary Material

Patterns of genomic (in)stability of interspecific yeast hybrids with diverse ancestries

**Devin P Bendixsen^1*^, David Peris^2,3^, Rike Stelkens^1^**

^1^Division of Population Genetics, Department of Zoology, Stockholm University, Svante Arrheniusväg 18B, 10691 Stockholm, Sweden

^2^Section for Genetics and Evolutionary Biology, Department of Biosciences, University of Oslo, N-0316 Oslo, Norway

^3^Department of Health, Valencian International University (VIU), 46002 Valencia, Spain

*** Correspondence:**Devin P Bendixsen
devin.bendixsen@zoologi.su.se


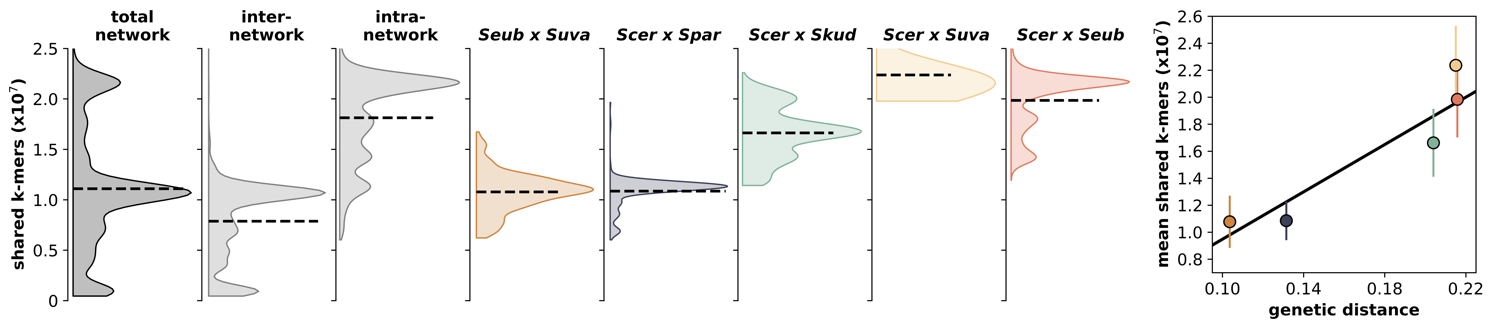


**Figure S1 Distributions of  shared k-mers for each hybrid cross with n=2.** Total network includes all pairwise k-mer comparisons both within and between hybrid crosses. Inter-network includes only k-mer comparisons between hybrid crosses. Intra-network includes all k-mer comparisons within each hybrid cross. Distributions for individual hybrid crosses include only k-mer comparisons with other genomes within the same hybrid cross. Dashed lines indicate the distribution mean. Correlation between mean shared k-mers and genetic distance between the two parental species. Vertical lines indicate the standard deviation of the mean.


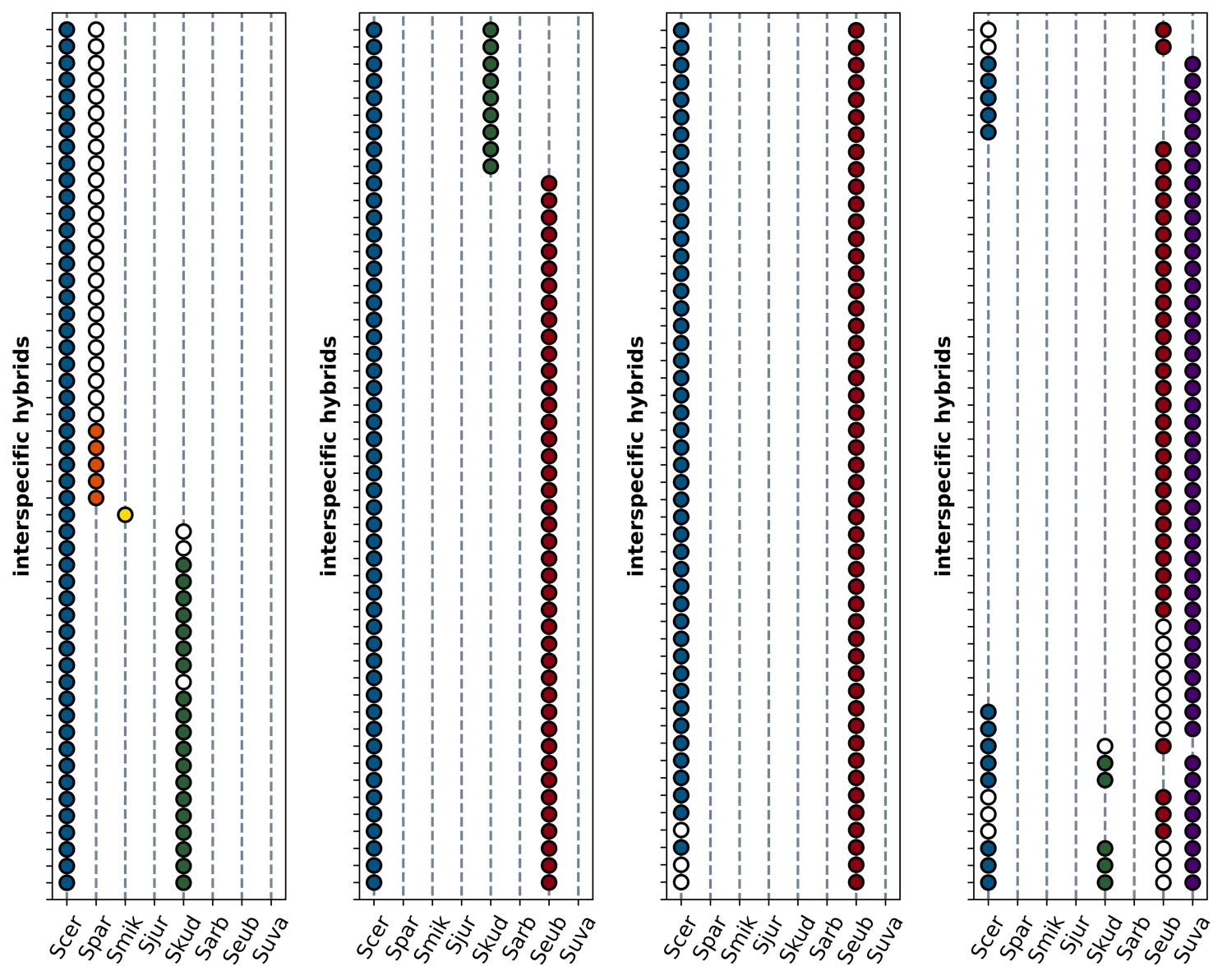


**Figure S2 Confirmation of species contributions using sppIDer.** Filled in circles indicate species contributions that were confirmed by producing a statistically significant positive residual in a χ^2^ test of the counts of reads that map to that species. Open white circles indicate species contributions that were described in the literature, however did not produce a positive residual, possibly suggesting only minor introgressions.


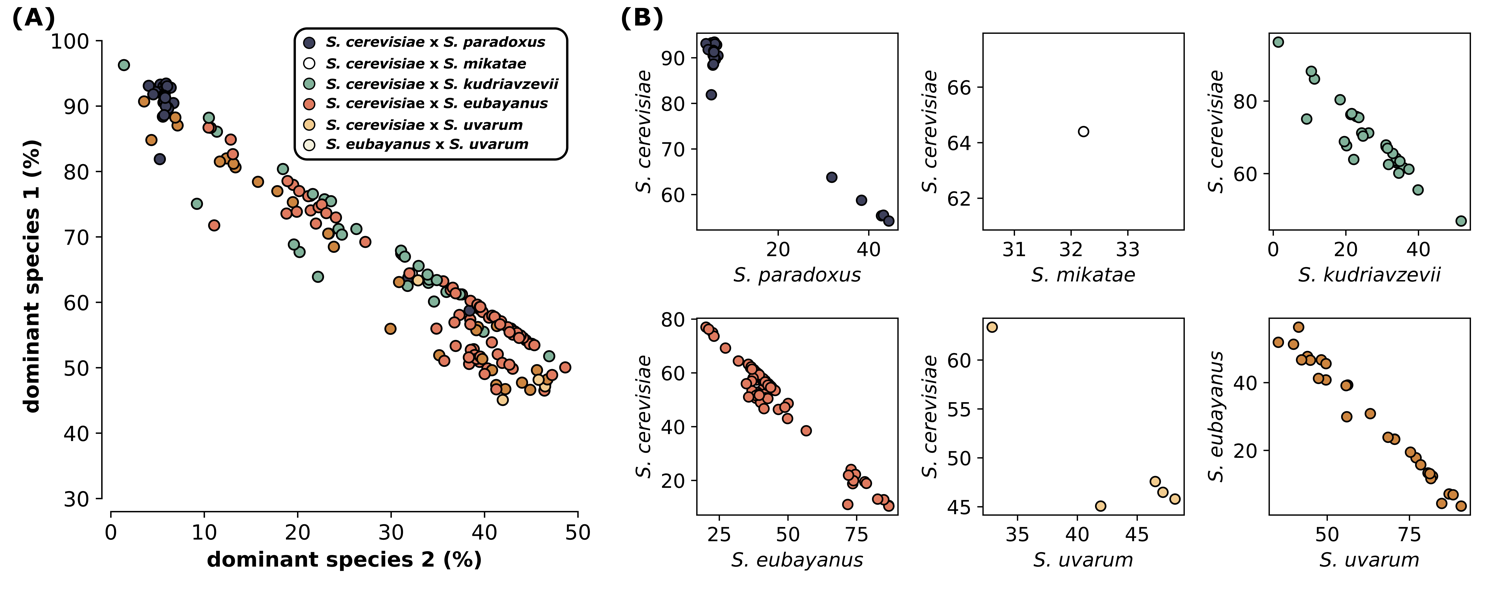


**Figure S3 Nuclear genomic contributions of two-parent interspecific hybrids. (A)** The relationship between the most dominant nuclear species and the second most dominant species depicted as percentage of reads mapping to the nuclear genome from each species. Data points are colored according to the hybrid cross. **(B)** The relationship between nuclear genomic contributions within each hybrid cross.


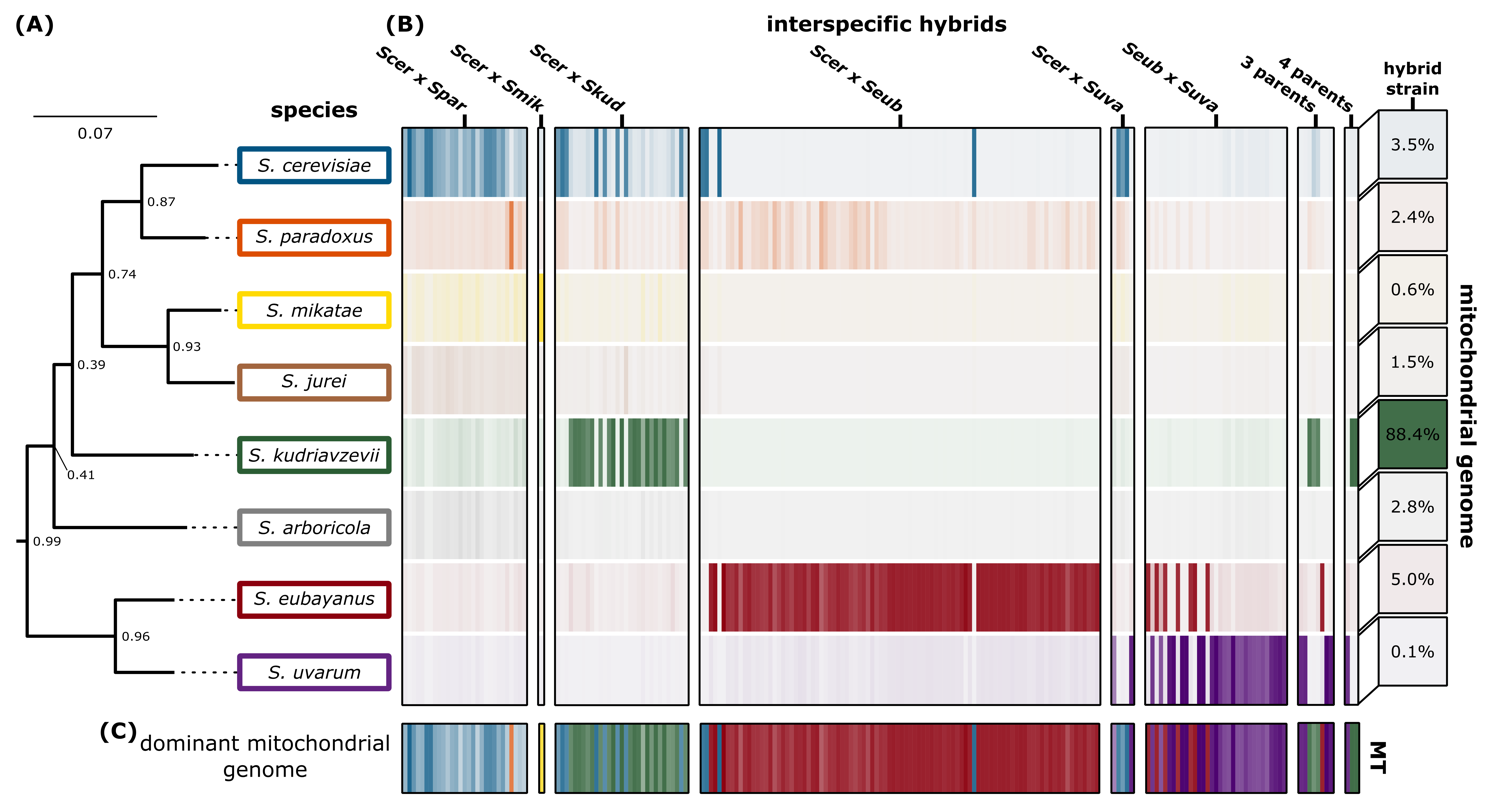


**Figure S4.** **Species contributions to mitochondrial genomes of interspecific hybrids. (A)** The phylogenetic tree indicates the phylogenetic relationship of the eight *Saccharomyces* species. The support values indicate the proportion of times that the bipartition is seen in each of the individual species tree estimates. Branch lengths represent the average number of substitutions per site across the sampled gene families. **(B)** Genomic contributions are calculated as the proportion of reads mapping to a representative of each species. The 204 interspecific hybrid strains span the horizontal axis, with the genomic contribution of each hybrid strain indicated. The intensity of each color indicates the genomic contribution for each species, with low (white) and high intensity indicating low and high genomic contributions, respectively. **(C)** The dominant mitochondrial genome within each hybrid strain is shown with similar coloring.

**
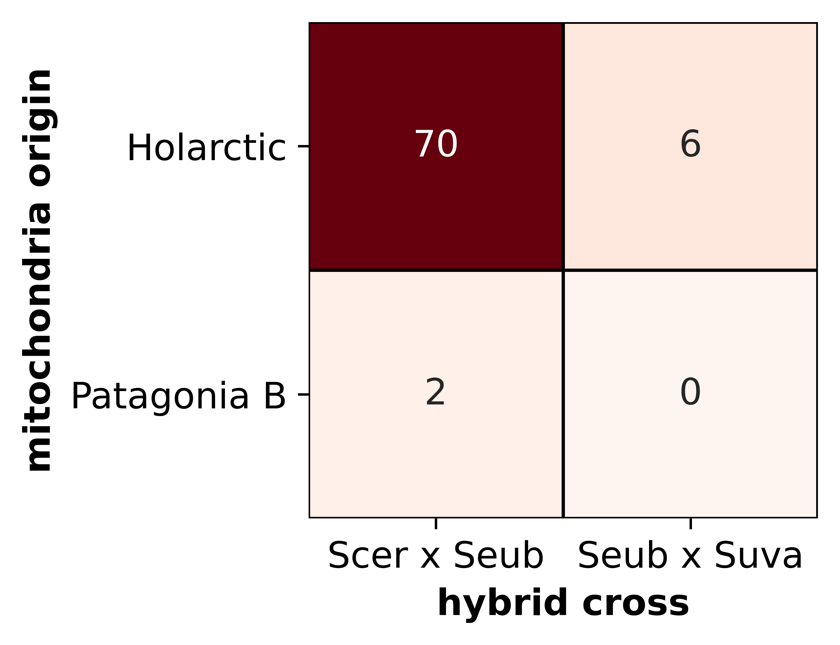
**

**Figure S5 Mitochondrial origin of hybrids that inherited *S. eubayanus* mtDNA.** For the two hybrid crosses with S. eubayanus, the prevalence of the two mitochondrial origins (Holarctic or Patagonia B) is depicted. The representative mitochondria used for holarctic was CDFM21L.1 and the representative mitochondria for Patagonia B was FM1318.


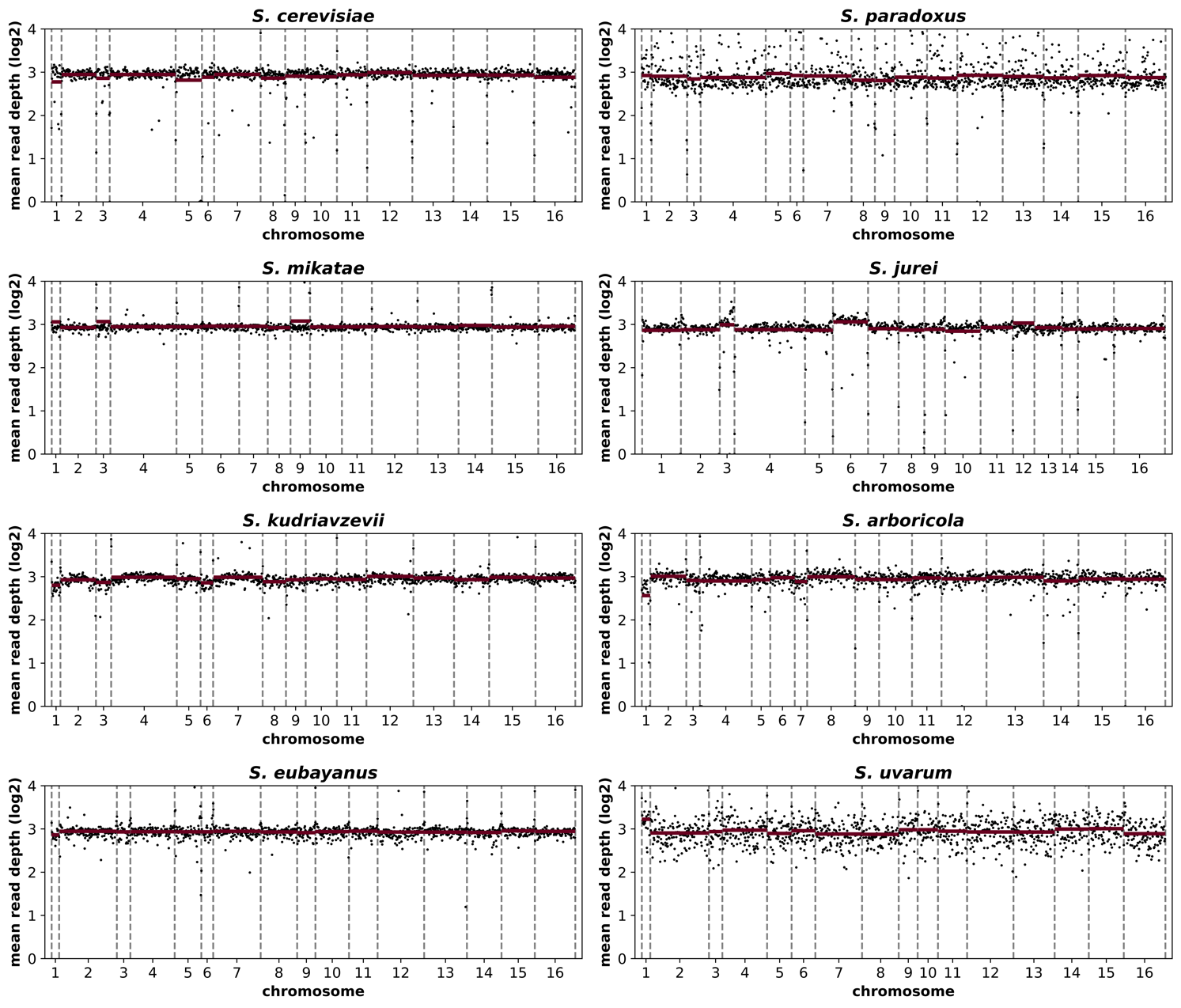


**Figure S6** **Sequencing read depth of *Saccharomyces* species.** Short read sequencing reads mapped to their respective genomes. Black dots indicate the read depth of ~10kbp windows. Dashed vertical lines separate chromosomes. Horizontal colored lines indicate mean chromosomal read depth.


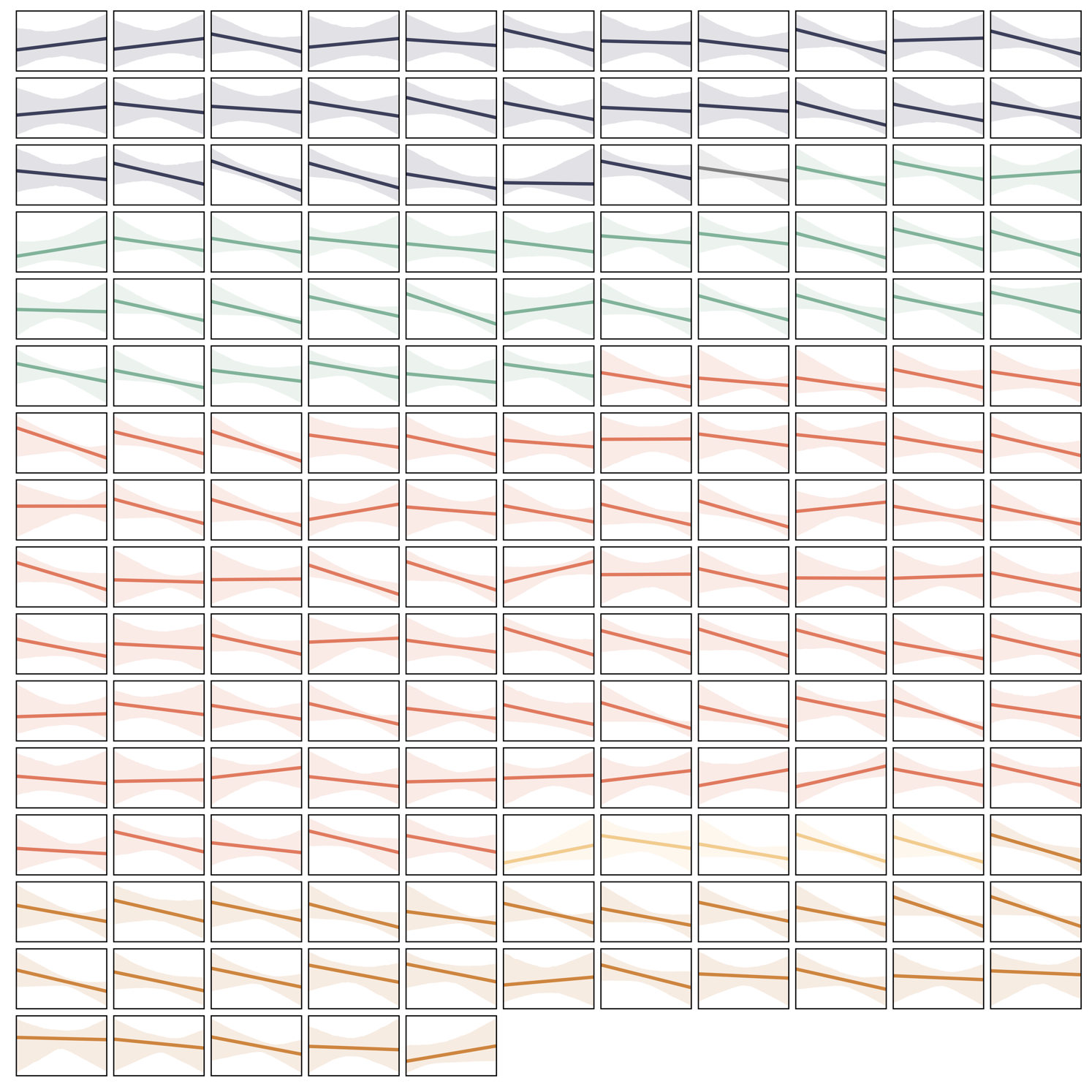


**Figure S7 Linear correlation between chromosome size and sequencing read depth**. Delta mean read depth is calculated as the difference (absolute value) between the total chromosomal read depth (species 1 + species 2) and the total genome read depth. Black circles indicate the means of independent chromosomes. The linear regression line for each hybrid genome with 95% confidence intervals is shown.


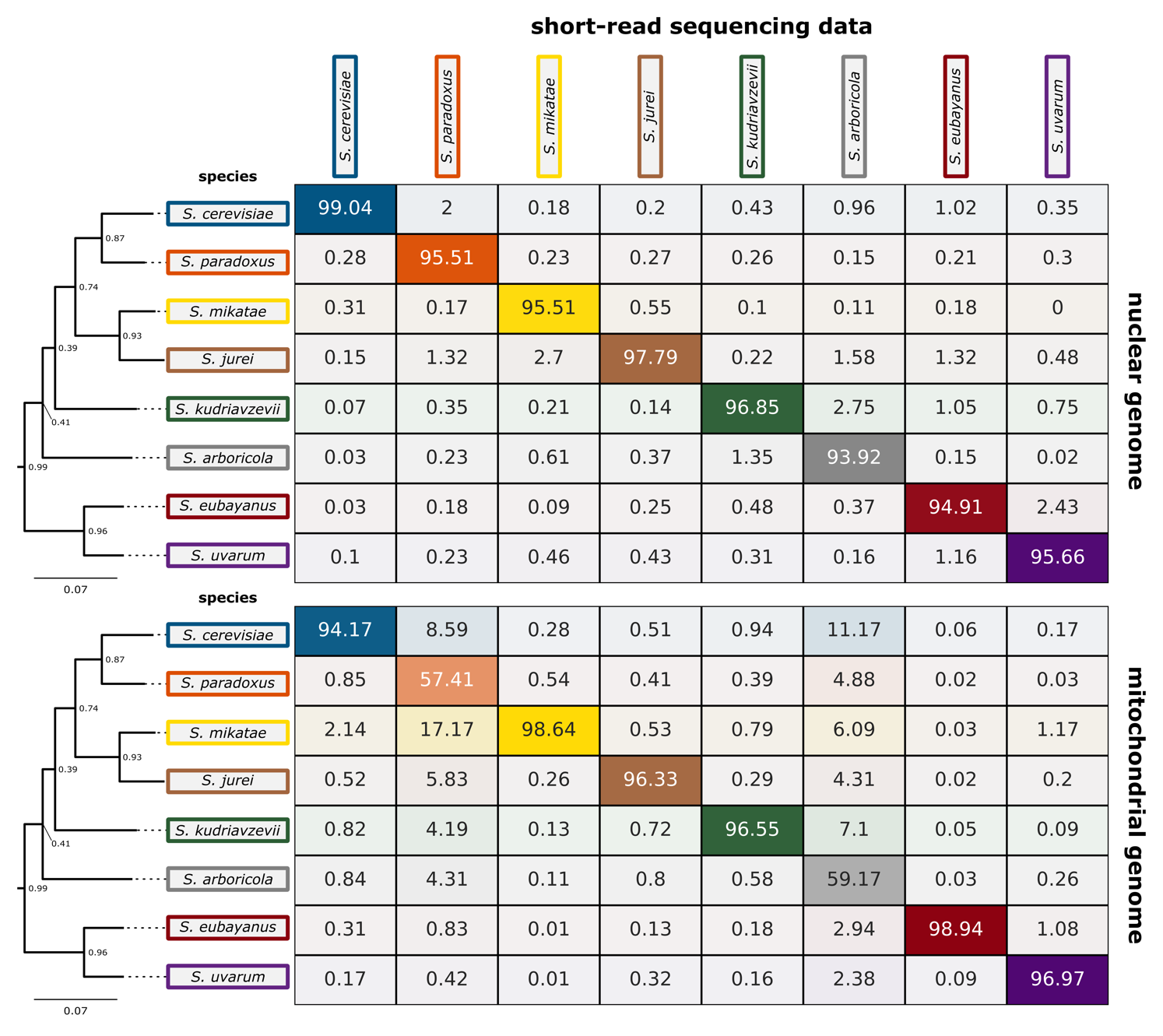


**Figure S8.** Confirmation of employing sppIDer and mitoSppIDer to detect species contribution of ‘pure’ species based on short-read sequencing data. Values indicate the percent of reads mapping to each assembled genome (either nuclear or mitochondrial) with nonzero MQ scores. The phylogenetic tree is taken from Figure 2.
